# Frontline Field Epidemiology Training Programs as a Strategy to Improve Disease Surveillance and Response 

**DOI:** 10.3201/eid2313.170803

**Published:** 2017-12

**Authors:** A. McKenzie André, Augusto Lopez, Samantha Perkins, Stephanie Lambert, Lesley Chace, Nestor Noudeke, Aissatou Fall, Biagio Pedalino

**Affiliations:** Centers for Disease Control and Prevention, Atlanta, Georgia, USA (A.M. André, A. Lopez, S. Perkins, S. Lambert, L. Chace, B. Pedalino);; African Field Epidemiology Training Network, Kampala, Uganda (N. Noudeke, A. Fall)

**Keywords:** epidemiology, training, surveillance, field epidemiology, outbreak, global health security, IDSR, FETP, Field Epidemiology Training Program

## Abstract

Since 1980, Field Epidemiology Training Programs (FETPs) have trained highly qualified field epidemiologists to work for ministries of health (MOH) around the world. However, the 2013–2015 Ebola epidemic in West Africa, which primarily affected Guinea, Liberia, and Sierra Leone, demonstrated a lack of field epidemiologists at the local levels. Trained epidemiologists at these levels could have detected the Ebola outbreak earlier. In 2015, the US Centers for Disease Control and Prevention (CDC) launched FETP-Frontline, a 3-month field training program targeting local MOH staff in 24 countries to augment local public health capacity. As of December 2016, FETP-Frontline has trained 1,354 graduates in 24 countries. FETP-Frontline enhances global health security by training local public health staff to improve surveillance quality in their jurisdictions, which can be a valuable strategy to strengthen the capacity of countries to more rapidly detect, respond to, and contain public health emergencies at the source.

Since their inception in 1980, Field Epidemiology Training Programs (FETPs) have been 2-year applied training programs focused on the practice of epidemiology in a mentored environment, with a focus on “learning by doing” (*1*). FETPs, which are adapted to the host country context, are designed to produce highly skilled epidemiologists who will work at the ministry of health (MOH) in each country to strengthen surveillance systems and respond to public health threats. The primary distinguishing characteristic of FETP is that most of the learning (≈75%) occurs in the field, at district- or national-level health offices. Trainees conduct fieldwork that simultaneously increases their capacity to apply epidemiologic concepts while strengthening the health system through the production of useful epidemiologic field products that provide information for decision making.

The Centers for Disease Control and Prevention (CDC) has a long history of providing technical assistance for FETPs (*1**,**2*), which were mostly modeled on CDC’s 2-year applied training through service program, the Epidemic Intelligence Service (EIS). Currently, CDC provides technical assistance to >65 FETPs throughout the world. These programs have been successful in strengthening epidemiologic and surveillance capacity at the national levels, but most programs did not address gaps at the subnational level.

Even before the West Africa Ebola outbreak, there were efforts to start a modified training program that could strengthen other levels of the public health system. In 2000, six Central American countries recognized a need for training of surveillance staff at the subnational level to collect quality surveillance data in a timely manner. The countries were part of a regional FETP that used a pyramidal approach to training; the 3 levels were dubbed basic, intermediate, and advanced and targeted local, regional, and national levels of the surveillance system (*3*). The curriculum of the 3-tiered training program was based on fundamental competencies of field epidemiology needed at each level of the surveillance system (*4*), with the purpose of improving the quality of surveillance and the ability to use surveillance data for action.

The Ebola outbreak in 2014 underscored the need for field epidemiology capacity at all levels of the healthcare system, in both affected and nonaffected countries in West Africa. Deficits in the public health surveillance system to identify cases and contacts at the local level and to respond in a timely manner were factors that contributed to the expansion and prolonged nature of the Ebola outbreak (*5*,*6*). Until the Ebola epidemic, most of the experience with FETP in Africa had been with the 2-year advanced-level program, which trained staff to work at national surveillance and disease control programs (*7*,*8*). This approach did not address the need to have adequately trained staff at the local level to detect outbreaks and respond appropriately.

In January 2015, in response to the urgent need for local capacity during the outbreak, CDC and several partners organized and conducted the emergency implementation of Surveillance Training for Ebola Preparedness (STEP). This program was designed to rapidly build surveillance capacity along the border districts and regions in the 4 countries sharing a land border with the heavily Ebola-affected countries, Guinea-Bissau, Senegal, Mali, and Côte d’Ivoire (*9*). The program was a simpler, shorter, and more focused version of the basic FETP, with an emphasis on the early identification of Ebola virus disease (EVD).

Shortly thereafter, longer-term planning to support surveillance capacity in the region began. Based on experience with basic FETP training and the successful emergency intervention of STEP, CDC developed a new strategy called FETP-Frontline. This training strategy targets public health staff working in surveillance at the local level to strengthen the capacity of countries to more rapidly detect, respond to, and contain public health emergencies at their source, preventing the spread of diseases and thereby enhancing global health security. FETP-Frontline development corresponded with the launch of the Global Health Security Agenda (GHSA). GHSA is an international collaboration between governments, international organizations, and implementing partners to help countries build the capacity to prevent, detect, and respond to public health threats from infectious diseases and achieve competencies necessary for compliance with the World Health Organization (WHO) International Health Regulations 2005 (IHR 2005) (*10*). Workforce development, which focuses on practical field-based epidemiology training, is 1 of the 11 Action Packages identified for strengthening to help countries to meet GHSA goals. This article describes the process and early results on the implementation of FETP-Frontline.

## Program Implementation

CDC staff visited each country and met with representatives of each MOH to describe the program and explore the value and feasibility of implementing FETP-Frontline. Upon agreement to launch the program, CDC staff, along with MOH colleagues, assessed the country’s training needs and priorities, gathering information from site visits and interviews with surveillance workers at multiple levels within the health system. Shortly thereafter, a 1- to 2-day implementation workshop was held with key stakeholders from relevant ministries within the country and key nongovernmental partners. During the meeting, leaders and stakeholders discussed strategic elements of program implementation, such as defining the subnational unit targeted for training and the personnel or job classes to be prioritized for training. In this article, we refer to the targeted administrative unit as the health district, even though the nomenclature varies across countries, because this is where data are first aggregated within the surveillance system. We also determined at the workshop possible sources of mentors to supervise participants in the field. Each country then developed a plan to cover all subnational units with >1 FETP-Frontline–trained person. 

In each country, a FETP team was established to work closely with the MOH, implementing partners, and the CDC country office to implement FETP-Frontline. Each team was led by a resident advisor, a senior-level epidemiologist who was either a CDC staff member or a contractor, usually from another country and a graduate of a 2-year FETP-Advanced. Other staff included a field coordinator, usually from the host country, who was most often a physician with experience in surveillance and epidemiology; and an administrator to assist with the logistics of program implementation. The resident advisor provided overall technical leadership for the program and worked closely with an identified MOH person to manage the program. The teams were often embedded within the MOH offices to facilitate planning and operation of the program.

### Trainees and Mentors

The persons targeted for the training were those responsible for collecting and analyzing health surveillance information, often called district surveillance officers. However, participants from other administrative levels were also eligible for training. In each country, the resident advisor and MOH counterparts identified mentors to provide onsite technical assistance to participants during the field stages. Mentors were ideally from within the MOH, with a ratio of 1 mentor to 5 participants. Once the strategic model was established, identified mentors were introduced to the FETP-Frontline curriculum and some basic adult-learning principles before the launch of the first training. The pretraining process typically took 3–6 months from the first meeting with the MOH to the first day of training for participants.

### Curriculum

The standardized curriculum and program schedule, incorporating both classroom workshops and on-the-job fieldwork, were originally developed in English and then translated into French and Portuguese to accommodate Francophone and Lusophone countries. Training materials also incorporated the Integrated Disease Surveillance and Response (IDSR) framework, which is used in 43 of 46 countries in the WHO Regional Office for Africa for disease surveillance and response reporting (*11*). In each country, the curriculum was then adapted to the country context, incorporating national reporting guidelines and practices. The classroom training is reinforced by the completion of field projects designed to help participants develop competencies related to specific job functions (Table 1).

**Table 1:** Fieldwork requirements as part of FETP-Frontline workshops*

Stage	Projects
Fieldwork stage 1, weeks 2–6: participants must complete both activities and present their findings at workshop 2	
Weekly surveillance report	Complete a weekly surveillance summary report based on health facility reports
	Record reporting timeliness and completeness; record key notifiable diseases
	Create graphs and figures that describe data
Data quality report	Examine the surveillance data collected in >3 different health facilities
	Conduct interviews with health facility staff; review log books, case forms, and posted bulletin boards
	Collect and review health facility weekly reports
	Complete a worksheet that organizes the findings from their data quality audit
Fieldwork stage 2, weeks 7–11: participants must complete 2 of the 4 activities and present their findings at workshop 3	
Case investigation report	Conduct a case investigation and interview a case or contact, using country-specific procedures when available
	Present details of the case investigation, including any public health action taken
Outbreak investigation report	Assist in outbreak investigation and develop an outbreak investigation report
	Maintain a rumor log book of suspected outbreaks
	Present report and findings
Expanded surveillance summary report	Continue creating weekly surveillance summary reports
	Analyze data to identify trends and gain a comprehensive view of the surveillance system
	Summarize the data and highlight trends or interesting characteristics at final workshop
Analysis of surveillance quality with recommendations	Critically examine a weakness that has been identified in the surveillance system during FETP-Frontline fieldwork
	Form a team with the surveillance personnel who are close to the issue in question; identify the critical causes of the problem
	Create a suitable solution to the problem that will lead to a direct improvement of the surveillance system

*FETP, Field Epidemiology Training Program.

**Figure 1:**
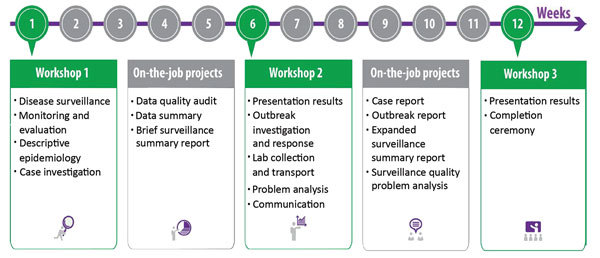
General program schedule showing the 3 classroom workshops (green boxes) and 2 field stages (gray boxes) in a standard Frontline Field Epidemiology Training Program curriculum.

The program schedule (Figure 1) for FETP-Frontline consists of an initial 5-day workshop introducing basic epidemiology principles and importance of disease surveillance. The participants then return to their regular job sites for 5 weeks. There, they receive onsite and remote mentoring from program staff to review local surveillance data and conduct a data quality audit around a priority disease in their coverage area. All Frontline FETP participants create a weekly surveillance report using real-world data derived from their home districts. The FETP resident advisors and mentors then guide the participants to aggregate and analyze the data at the district level. The participants return for a second 5-day workshop to present their work and receive feedback from the staff and their peers on their projects. During the second workshop, participants learn how and when to conduct field investigations and how to effectively communicate results. Participants then return to the field for the second 5-week field stage to put in practice what they have learned under the guidance of the mentors and to complete 2 of 4 possible field activities: conducting a field investigation to confirm or rule out a reportable disease, participating in an outbreak investigation, developing an expanded surveillance summary report, or completing an analysis of surveillance quality with appropriate recommendations. In the third workshop, participants present their final projects and receive a certificate of course completion cosigned by MOH and CDC representatives. At the end of each module and in between cohorts, the technical staff conducted internal evaluations. Participant feedback is gathered through questionnaires. Program staff are encouraged to review the feedback and work with the MOH to tailor the curriculum materials and training schedule as needed.

## Results (Status)—Principles of Frontline FETP Implementation

**Figure 2:**
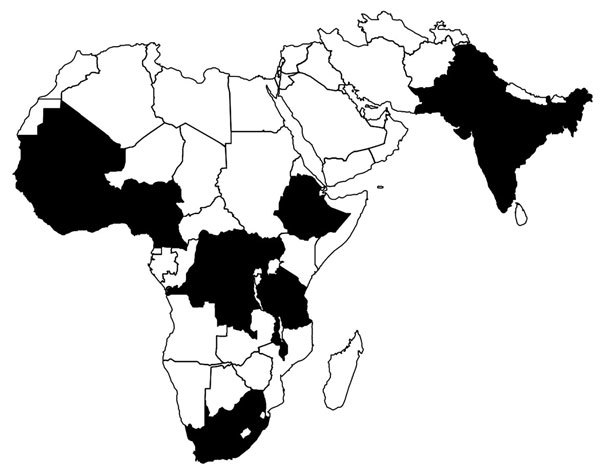
Geographic coverage of Frontline Field Epidemiology Training Programs established (black), July–December 2016.

**Figure 3:**
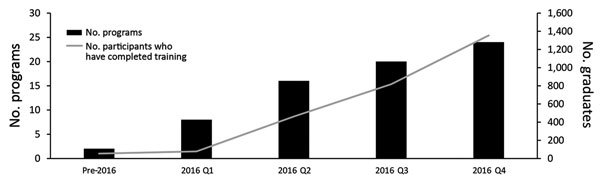
Frontline Field Epidemiology Training Programs launched and cumulative number of participants trained by quarter (Q) of program launch through Q4 2016. Quarter of launch is defined by the date of the first classroom session.

The first FETP-Frontline cohort began in Tanzania in July 2015. All the FETPs -Frontline that started in 2015 and 2016 were in Africa and southern Asia, with a heavy concentration in West Africa (Figure 2). The FETP-Frontline underwent a rapid expansion across these countries, with most programs launching their first cohort during the first 6 months of 2016 (Figure 3).

From the program’s launch in July 2015 through the end of 2016, a total of 1,354 persons completed FETP-Frontline training (Figure 3). Participants were almost all MOH employees and represented a variety of backgrounds: data managers, nurses, physicians, environmental health officers, veterinarians, laboratorians, and public health officials. The proportion of districts in each country with >1 FETP-Frontline–trained surveillance officer has expanded steadily. Four countries (Sierra Leone, Guinea-Bissau, Liberia, and Senegal) achieved complete or near-complete district-level coverage by the end of 2016 (Table 2).

**Table 2:** Proportion of districts or other designated subnational health unit with >1 trained FETP-Frontline graduate for 24 participating countries, 2016*

Country	Total no. districts	% Districts with >1 Frontline FETP graduate			
		Q1	Q2	Q3	Q4
Sierra Leone	14	0	100	100	100
Guinea-Bissau	11	0	73	100	100
Liberia	90	0	57	76	76
Senegal	76	0	53	53	74
Côte d'Ivoire	82	NA	15	15	29
Benin	82	0	28	28	28
Nigeria	774	NA	14	23	26
South Africa	52	NA	0	4	17
Cameroon	178	NA	8	8	15
Ghana	216	NA	13	13	13
Uganda	112	NA	4	13	13
Tanzania	169	NA	7	12	12
Burkina Faso	70	NA	0	11	11
Bangladesh	490	4	4	4	9
Malawi	29	NA	3	3	7
Democratic Republic of the Congo	517	NA	3	3	3
India	687	2	2	2	2
Ethiopia	880	NA	NA	0	0
Mauritania	55	NA	NA	0	0
Gambia	43	NA	0	0	0
Guinea	33	NA	NA	NA	NA
Mali	49	NA	NA	NA	NA
Pakistan	149	NA	NA	NA	NA
Togo	40	NA	NA	NA	NA

*Most programs target participants at the district level or its equivalent. This is typically the first surveillance level at which data are aggregated (immediately above the health facility level). FETP, Field Epidemiology Training Program; NA, no coverage data available because the first cohort of FETP-Frontline had not yet graduated; Q, quarter.

Weekly surveillance data were collected from 3 countries for the duration of FETP-Frontline. The timeliness of surveillance reporting, defined as the proportion of weekly surveillance reports delivered to the district level by a predetermined deadline, from these 3 programs increased from an average timeliness rate of 33% in week 1 to 96% in week 12. An example can be seen in the reported timeliness data from the first cohort in the Benin program, in which the average reported timeliness went from 37% on-time to 85% on-time (Table 3).

**Table 3:** Effect of FETP-Frontline training on timeliness of surveillance reporting by health district, Benin, epidemiologic weeks 25–36, 2016*

Health district	Epidemiologic week														
		Workshop 1		Fieldwork 1							Workshop 2		Fieldwork 2		
	25	26		27	28	29	30	31	32		33		34	35	36
NIKKI	94	94		88	56	31	31	38	38		44		75	94	94
SO-AVA	56	56		56	78	100	100	100	100		100		100	100	100
PEV d'Abomey-Calavi	25	25		38	50	63	75	75	88		100		100	100	100
Save	0	0		42	83	83	92	100	100		100		100	100	100
Zagnanado	25	0		0%	50	100	100	100	100		100		100	100	100
Malanville	100	100		100	100	100	100	100	100		100		100	100	100
Allada	25	25		50	75	100	100	25	50		25		75	100	75
Cotonou 6	NR	NR		NR	NR	NR	NR	50	50		100		75	100	100
Aguégués	0	0		0	0	100	100	100	100		100		100	100	100
Pobe	67	83		100	83	83	83	100	100		100		100	100	100
Abomey-Calavi	25	25		38	50	63	75	75	88		100		100	100	100
Ze	50	75		100	100	100	100	100	100		100		100	100	100
Sèmè-Podji	30	20		30	40	60	80	90	90		100		100	100	100
Ifangni	9	27		9	9	9	36	9	9		9		9	9	45
Adja-Ouèrè	100	100		100	100	100	100	100	100		100		100	100	100
Adjarra	14	29		43	43	57	57	71	57		71		57	57	57
Tchaourou	31	54		46	46	46	62	100	100		100		100	100	100
Perere	0	0		27	36	36	36	45	36		36		45	18	36
Kalale	27	27		40	53	87	93	67	80		87		87	87	93
Cotonou V	0	0		0	0	75	75	75	75		75		75	75	75
Segbana	100	100		100	100	100	100	100	100		100		100	100	100
Cotonou I and IV	0	0		0	0	0	0	0	0		0		0	0	0
Weekly average	37	40		50	55	71	76	74	75		79		82	84	85

*Timeliness is defined as the percentage of reports from the health facility level that are delivered to the district by a predetermined deadline (typically weekly). FETP, Field Epidemiology Training Program; NR, not reported.

FETP-Frontline participants have used their training to identify gaps and promote change in the public health systems in which they work. Guinea-Bissau FETP-Frontline participants made policy recommendations to improve the way in which dog bites are tracked, in terms of follow-up with rabies testing, and to improve data confidentiality and protection for patients. In The Gambia, under the resident advisor’s guidance, members of the first cohort created recommendations for improving the surveillance system; among these were appointing district surveillance officers where there were none previously, training new staff in basic epidemiology, implementing and revising protocols to match IDSR recommendations, and including private health clinics in the national surveillance strategy. Liberia realized a need to appoint surveillance personnel between the community and regional levels.

In Côte d’Ivoire, only 4 of the 36 participants in the first 2 cohorts had ever conducted a field investigation before the training; upon program completion, 20 had conducted a field investigation with the assistance of a field mentor. Investigations included suspected cases of yellow fever, measles, and rabies (Table 4). In Liberia, participants conducted outbreak investigations on conditions such as food poisoning, suspected acute flaccid paralysis, and measles. In the Democratic Republic of the Congo, participants investigated typhoid fever, yellow fever, and cholera outbreaks. In Benin and Burkina Faso, program participants have mobilized from their home districts to respond to outbreaks in other parts of the country, serving as a trained, in-country pool of epidemiologists from which to draw during emergencies. In Côte d’Ivoire, Senegal, and Togo, where training has included participants from both the human and animal health sectors, trainees have worked together to conduct coordinated joint investigations to combat rabies.

**Table 4:** Field products completed by the first 2 cohorts of FETP-Frontline participants in Côte d’Ivoire, May–December 2016*

Field product	Total no.
Expanded weekly surveillance report	36
Topics for the problem analysis report	17
Late- reporting or underreporting of surveillance data	6
Nonapplication of case definitions	3
Poor community notification of cases	2
Inadequate local surveillance data analysis	2
Underreporting of maternal deaths	2
Other	2
Conditions identified for field investigation report	20
Suspected case of yellow fever	6
Suspected case of measles	4
Other vaccine-preventable disease	4
Gastrointestinal illness/diarrhea	3
Rabies	2
Suspected case of hemorrhagic fever	1
Cluster of acute respiratory illness	1

*FETP, Field Epidemiology Training Program.

## Discussion

As countries address gaps in surveillance and begin to develop the core capacities for surveillance and response as set by the IHR 2005, they will need to ensure that there is capacity at the local level “to detect unusual public health events, to report key epidemiological information to relevant intermediate and national authorities, and to immediately implement primary control measures” (*12*). The FETP-Frontline was initiated as a response to identified gaps in surveillance and response capacity at the local level. In many developing countries, district-level surveillance officers have historically only passed information on to the national level, without taking the opportunity to analyze the data locally or respond immediately. These missed opportunities can contribute to delays in disease recognition and timely interventions. By tailoring a training program and field products to the routine responsibilities and expected job duties of a district surveillance officer, participants develop relevant and practical competencies in field epidemiology.

FETP-Frontline has targeted the district level for training because, quite simply, this is where the action is. In most countries, the district level is the point at which surveillance data first enter the formal public health system and also the point at which data are aggregated and can be analyzed to detect abnormalities and represents the first opportunity to mount a public health intervention. Preliminary data from FETP-Frontline have shown improvements in local detection and response capacity within weeks of initiating the training. This capacity can be seen in the local functioning of the public health surveillance system. There have been improvements in the timeliness of surveillance reporting and an increase in field activity that result in quicker identification of diseases in the community. The purpose of FETP-Frontline is not only to improve the timeliness of the surveillance data that are collected but also to improve the quality of the data and to promote critical thinking by district-level surveillance officers who are responsible for the data. In conducting data quality analysis, trainees identify gaps and propose recommendations to improve surveillance in their locales. During the third workshop, higher-level members of the surveillance system are invited to attend the presentations and react to some of the findings, ensuring that the problems identified during the field work are brought to the attention of MOH leadership. Some of the recommendations formulated by FETP-Frontline participants have already led to local changes in surveillance systems such as the adoption and utilization of rumor logs, increased distribution of standardized case definitions for diseases under surveillance, and increased emphasis on surveillance data during monthly district management meetings.

The successful implementation of FETPs-Frontline occurred simultaneously across several countries and demonstrated that a large-scale, multicountry capacity-building program could be implemented quickly with external support and country engagement. This effort did not take staff away from their jobs in-country and provided benefits in a short timeframe by addressing actual problems at individual work sites. However, for the program to be sustainable, countries will ultimately have to take on the technical and logistical leadership of the program. The implementation of FETPs-Frontline is not without challenges. This initiative was greatly supported by the CDC and several partners including WHO, the African Field Epidemiology Network (AFENET), Training Programs in Epidemiology and Public Health Interventions Network (TEPHINET), and the Defense Threat Reduction Agency (DTRA). Because the FETP-Frontline model is continuing education for existing public health personnel, it requires the involvement and commitment of the host country’s MOH. During the training, each participant received >1 day of onsite mentoring and supervision during each of the 2 field stages. Mentorship in the field requires both financial and technical resources. The costs for implementing FETP-Frontline varied from US $5,000 to $8,000 per student (data not shown) based on many factors, including the existence of locally trained personnel.

Several countries had difficulty identifying professionals with the appropriate skills and experience in field epidemiology who could devote the time required to mentor participants. Several strategies were used to address this gap, including providing an orientation on effective mentoring techniques for staff, fully training a small group of central-level candidates in the first cohort to familiarize them with the field-based training approach and then having them serve as mentors for later cohorts, and engaging mentors from outside the country for the first few cohorts.

There is a concern that, once trained, graduates may leave their posts for better opportunities outside the public health system. A few countries have addressed this issue through the following mechanisms: making participation in the program contingent upon staying in that position for a set period, setting an upper age limit for participants so that newly trained staff will not retire shortly after the course, and designating new and more appropriate positions for those who are trained in FETP-Frontline. FETP-Frontline will need to continue until there is a critical mass of trained personnel representing each district or other identified subnational unit in every country. MOHs are responsible for continuing to support the training to address staff turnover and to make available the resources for the field activities of effective public health surveillance.

Although comparing the outcomes of FETP-Frontline implementation between countries is complicated due to the wide variability in public health systems, there are important lessons and implications for other countries from each implementation. Currently, standard indicators across programs are in development. The national IDSR indicators and the results of efforts such as the independent Joint External Evaluation process will enable countries to track progress in detecting and responding to emergencies (*13*). It is likely that other countries can learn from the lessons in FETP-Frontline implementation we have described and embark upon efforts to launch the program for themselves.

In-service FETP-Frontline training can be an effective strategy to improve the functioning of a public health surveillance system in a short time with immediate benefits. Trainees are working in their home districts, analyzing their own data, addressing their local health priorities, and identifying ways to better detect and respond to public health emergencies given their unique local constraints. By empowering actors to analyze and intervene at the district level, the program helps decentralize some of the initial analysis and decision making, which leads to more accurate communication within the system and a timelier public health response. In some countries, the veterinary and laboratory sectors were included in training cohorts to foster local cross-sector collaboration and a One Health approach to surveillance and response activities. This initiative should be viewed not as a training program but as part of a larger workforce development strategy to improve a country’s local surveillance and response capacity that complements FETP training activities at the intermediate and advanced levels. Participants who have completed the training are contributing to enhanced global health security by being able to detect outbreaks sooner, respond faster, and, through quick response, limit the spread of infectious disease outbreaks at the source.
